# Exploratory Analysis on the Use of Statins with or without n-3 PUFA and Major Events in Patients Discharged for Acute Myocardial Infarction: An Observational Retrospective Study

**DOI:** 10.1371/journal.pone.0062772

**Published:** 2013-05-06

**Authors:** Alejandro Macchia, Marilena Romero, Antonio D’Ettorre, Gianni Tognoni, Javier Mariani

**Affiliations:** 1 Laboratory of Pharmacoepidemiology, Consorzio Mario Negri Sud, Santa Maria Imbaro, Chieti, Italy; 2 GESICA (Grupo de Estudio de Investigación Clínica en Argentina) Foundation, Buenos Aires, Argentina; Consorzio Mario Negri Sud, Italy

## Abstract

**Background:**

Combined treatment (CT) with statins and polyunsaturated fatty acids (n-3 PUFA) resulted in a reduction of death and major cardiovascular events when administered after a myocardial infarction (MI). However, recent data suggests that CT may be ineffective because patients are currently treated aggressively and the risk may not be further decreased. We aimed to study the prevalence and the results on major outcomes with CT among patients discharged with a MI in Italy.

**Methodology/Principal findings:**

Retrospective cohort study that used linked hospital discharge, prescription databases and vital statistics containing information on 14,704 patients who were discharged for MI between 1/2003 and 12/2003 in 117 hospitals in Italy. All analyses were time-dependent and adjusted for major confounders. Sensibility and paired matched analysis were conducted to further verify main results. A total of 11,532 (78.4%) filled a prescription for a statin. Of these, 4302 (37.3%) were on CT. There were 45,528 patients/years of follow-up. As compared with statins alone, CT was associated with an adjusted higher survival rate (HR = 0.59 [0.52–0.66], p<0.001), survival free of atrial fibrillation (HR = 0.78 [0.71–0.86], p<0.001) and survival free of new heart failure development (HR = 0.81 [0.74–0.88], p<0.001), but not with re-infarction (HR = 0.94 [0.86–1.02], p<0.127). Clinically this means that between 2 to 3 fewer events for each 100 patients/year were obtained in the group under CT.

**Conclusions/Significance:**

Among a representative sample of patients discharged with MI in Italy, we observed clinically significant synergism between the effects of statins and n-3 PUFA for most cardiovascular outcomes, including all cause mortality.

## Introduction

The landmark GISSI Prevenzione trial demonstrated that an oral supplementation with 1 daily gram of n-3 PUFA was associated with a decreased mortality and improve outcomes in post-MI patients, mostly by a reduction in cardiac arrhythmias [1], [2]. However, recent evidence failed to replicate these results for reasons that remain speculative [3]–[8]. One of the most cited hypothetical reason inform that within the last 10 years, patients become more aggressively treated with statins, and that fish oil may have a ceiling effect on top of the compounds. In fact, combination therapy with n-3 PUFA and statins were not formally tested in this type of population and epidemiological data are also relatively scarce.

Following the approval in Italy and in the other European countries of n-3 PUFA as a recommended component of secondary prevention, n-3 PUFA and statins became part of post-MI strategies, with the suggestive hypothesis that their combination could be complementary, not because of an additive role on the lipid control, but via the sum and/or the interaction of independent mechanisms of action [3], [9], [10]. Further, in the factorially designed GISSI-HF study, neutral effects were found for rosuvastatin while n-PUFA proved efficacious in decreasing mortality, with further evidence of an antiarrhythmic component [11].

Within this context, we aimed to explore the outcomes of a large and contemporary population prescribed with statins and to study major outcomes accordingly to if they were or were not also prescribed with n-3 PUFA.

## Methods

A cohort of up to 14,704 patients discharged alive following their first MI (ICD IX, code 410) between January 1,2003 and December 31, 2003 and followed for 4 years, was established by linking the administrative databases of drug prescriptions and hospitalizations of 117 Coronary Care Units (CCUs) of the NHS covering on overall population of 7,5 millions across 22 Health regions of Italy.

The methodology for the quality control of the clinical data, and the reliability of their linkage also with the registries of mortality, had been extensively tested and validated [12], [13]. Hospital discharge records include information on primary diagnoses and up to 5 coexisting conditions, performed procedures, date of admission, discharge and in-hospital death.

Using encrypted affiliation numbers, this cohort was linked with the drug claims database which provides the community prescriptions reimbursed by the NHS with drugs coded according to the Anatomical Therapeutic Chemical (ATC) Classification [14] and qualified with respect to dosages, date of first prescription and duration of exposure.

### Ethics

The analysis complied strictly with the national Italian regulations for the full protection of the privacy rights of the subjects included in the databases.

According to the Italian law, no ethical approval is required to perform this type of analysis, so no committee was involved in this study.

Written informed consent was not taken because Italian regulation states that for this type of analysis this is not due.

### Cohort Definition

The study cohort is represented by all patients consecutively discharged alive in the aforementioned period, with a primary diagnosis of MI and who fulfilled a prescription of statins with or without n-3 PUFA during the first 30 days of hospital discharge. This cohort was then followed up for up to 1,461 calendar days (4 years), after the date of hospital discharge.

The 12-months period preceding the data of the index event was analyzed to identify several cardiovascular and non-cardiovascular conditions as documented by hospitalizations and/or chronic exposures to pharmacological treatments used as identifiers of underlying clinical conditions. Identified cardiovascular comorbidities included the presence of previous stroke, transient ischemic attack (TIA), peripheral vascular disease, embolic episode, atrial fibrillation, hypertension, congestive heart failure (CHF), previous infarction and diabetes, as reported elsewhere [12], [13]. Non-cardiovascular conditions included the identification of malignancy, previous hospitalization for major bleeding, chronic obstructive pulmonary disease (COPD) and depression.

Considering that patients could switch back and forward from the baseline prescription group during the 4-year follow up period, we created a time-dependent variable to assess the follow-up group of adjudication every 3 months period [15]–[17]. Patients who switched group during the follow-up period were analyzed accordingly.

### Statistical Analysis

Unadjusted mortality throughout 4 year of follow-up for each group was summarized by using Kaplan–Meier curves and compared by using the log-rank test. To account for differences in follow-up and to control for differences in baseline characteristics among groups, a multivariable time-dependent Cox proportional hazards model [15]–[18] was used. In all Cox models, the associations between groups and all outcomes were adjusted for age and sex, as well as for the presence of all the clinical cardiovascular (CV) conditions and non-CV comorbidities. The regression model included also both in-hospital procedures (cardiac catheterization, percutaneous coronary intervention, coronary artery bypass graft surgery) and discharge medications (β-blockers, aspirin, calcium channel blockers, diuretics and antithrombotic agents). The associations of treatment groups and all outcomes took in consideration the last group of treatment for each patient.

Forced-entry regression was used to include these variables in all multivariable models in order to adjust the between-group comparisons for potential confounders. For the analysis of survival free of atrial fibrillation, survival free of stroke and survival free of heart failure we only considered those patients who did not have these conditions at baseline.

We used a fixed date as a censoring point for patients still alive, which enabled us to have up to 4 year of follow-up information for all patients. As in our mortality analyses, censoring occurs only at this date.

All mortality and major outcomes hazard ratios for all groups of patients were tested including time-dependent variables, to verify that the potential differences in the exposure time and switch between groups do not affect results.

### Sensitivity Analysis

To assess the robustness of our findings about potential confounding by indication, we performed sensitivity analyses. Firstly, we reanalyzed data within certain subgroups, including elderly patients (≥70 years), and those with other several conditions known to be indicators of increased risk (diabetes, hypertension, previous MI, previous heart failure, previous revascularization) to verify if the associations of major outcomes with treatments remained similar. Similarly we explored if the effect of combination therapy was similar among patients optimally treated with antiplatelets, β-blockers and ACE/ARB inhibitors and those who were not optimally treated.

Additionally, to further assess consistency, we conducted a propensity score matching analysis. For this purpose, we constructed a propensity score (i.e. the probability of being treated given a combination of confounders) for the exposure to combination therapy using a logistic regression (LR) model, and then we matched these patients to those exposed to statins only, with a difference in propensity scores less to 0.01 in 1∶1 ratio. The association between combination therapy and outcome in propensity-matched pairs was assessed using McNemar’s test.

All reported p values are two tailed, and a p value less of 0.05 was considered as indicating significant difference. We did no adjustments for multiple comparisons. All analyses were conducted using SPSS version 16.0 for Windows (SPSS Inc., Chicago, Ill).

## Results

### Population

Up to 78.4% of the 14,704 patients discharged post-MI were prescribed statins with or without n-3 PUFA within the first 30 days of follow-up.

Table 1 describes the demographic, clinical and hospital characteristics of patients according to their exposure to the treatments of interest at discharge.

**Table 1:** Summary of main baseline characteristics.

Variable	All patients	Statins	Statins plus n-3 PUFA		P value
N	11,532	7,230	4,302		–
Age, mean (SD)	66.7 (10.8)	68.5 (10.4)	63.8 (10.7)		<0.001
Male sex, n (%)	8,063 (69.9)	4,820 (66.7)	3,243 (75.4)		<0.001
*Previous cardiovascular risk factors and cardiovascular conditions*					
Diabetes, n (%)	3,121 (27.1)	1,957 (27.1)	1,164 (27.1)		0.990
Hypertension, n (%)	7,944 (68.9)	5,177 (71.6)	2,767 (64.3)		<0.001
Previous MI, n (%)	538 (4.7)	322 (4.5)	216 (5.0)		0162
Previous CHF, n (%)	1,579 (13.7)	1,084 (15.0)	495 (11.5)		<0.001
Previous Stroke, n (%)	258 (2.2)	195 (2.7)	63 (1.5)		<0.001
Previous AF, n (%)	817 (7.1)	597 (8.3)	220 (5.1)		<0.001
Peripheral vascular disease, n (%)	749 (6.5)	484 (6.7)	265 (6.2)		0.260
Previous CABG, n (%)	100 (0.9)	62 (0.9)	38 (0.9)		0.885
Previous PTCA, n (%)	477 (4.1)	281 (3.9)	196 (4.6)		0.081
*Previous non-cardiovascular conditions*					
COPD, n (%)	1,338 (11.6)	933 (12.9)	405 (9.4)		<0.001
Malignancy, n (%)	324 (2.8)	229 (3.2)	95 (2.2)		0.003
Depression, n (%)	363 (3.1)	258 (3.6)	105 (2.4)		0.001
*Cardiovascular prescriptions at discharge*					
Beta-blockers, n (%)	9,104 (78.9)	5,469 (75.6)		3,635 (84.5)	<0.001
ACEi/ARB, n (%)	10,238 (88.8)	6,347 (87.8)		3,891 (90.4)	<0.001
Aspirin, n (%)	10,462 (90.7)	6,459 (89.3)		4,003 (93.0)	<0.001
Statin agent, n (%)					0.502
Atorvastatin	5,084 (44.1)	3,218 (44.5)		1,866 (43.4)	
Simvastatin	4,004 (34.7)	2,502 (34.6)		1,502 (34.9)	
Rosuvastatin	528 (4.6)	334 (4.6)		194 (4.5)	
Other	1,916 (16.6)	1,176 (16.3)		740 (17.2)	

As compared with the patients treated only with statins, patients who received combination therapy were significantly younger, with a higher proportion of males, with a lower prevalence of cardiovascular and non-cardiovascular comorbidities at baseline and a higher probability of receiving aspirin, beta blockers and ACE inhibitors at hospital discharge.

Table 2 shows the unadjusted analyses of the outcomes observed over the 45,528 patients/years of follow-up (27,846 for those exposed to statins and 17,682 for those exposed to statins plus n-3 PUFA).

**Table 2:** Unadjusted incidence rate for major outcomes in patients treated with statins alone or statins plus n-3 PUFA*.

Outcomes	All	Statins	Statins+n-3 PUFA	Incidence rate difference
Overall mortality (n = 1,591)	3.5	4.5	2.0	−2.5 (−2.2 to −2.9) p<0.001
Death or reinfarction (n = 2,545)	6.1	6.8	5.1	−1.7 (−1.2 to −2.1) p<0.001
Death or atrial fibrillation (n = 2,458)	5.8	6.9	4.0	−2.9 (−2.5 to −3.4) p<0.001
Death or new heart failure (n = 2,439)	5.8	6.7	4.2	−2.5 (−2.1 to −2.9) p<0.001
Death of stroke (1,907)	4.3	5.4	2.6	−2.8 (−2.4 to −3.1) p<0.001

* Number are expressed as incidence per 100 patients-year of follow-up.

### Overall Mortality

Over the four years of follow-up, there were 1,591 fatal events, representing an overall death rate of 3.5 per 100 patients/year, 4.5 per 100 patients/year and 2.0 per 100 patients/year, for patients receiving statins and statins plus n-3 PUFA, respectively (incidence rate difference −2.5 per 100 patients/year; 95% CI −2.2 to −2.9; p<0.001).

### Other Outcomes

Overall, 2,545 patients died or had a reinfarction during follow-up (incidence rate 6.1 per 100 patients/year). As compared with patients prescribed with statins alone, those prescribed with statins plus n-3 PUFA had a statistically significant lower rate of death or reinfarction (6.8 per 100 patients/year versus 5.1 per 100 patients/year, respectively; incidence rate difference −1.7 per 100 patients/year, 95% CI −1.2 to −2.1; p<0.001).

As detailed in Table 2, closely similar results were observed for the combined end-point of mortality and AF, as well as of mortality and CHF and mortality and stroke.

### Adjusted Analyses

**Figure 1:**
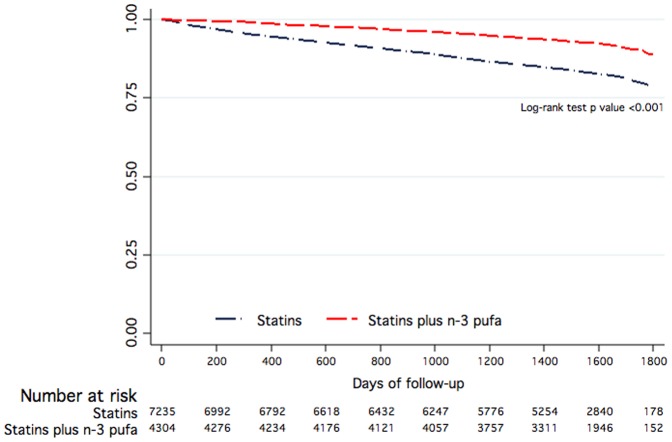
Kaplan-Meier survival curves of the effects of statins plus n-3 PUFA as compared with statins alone on overall survival.

**Figure 2:**
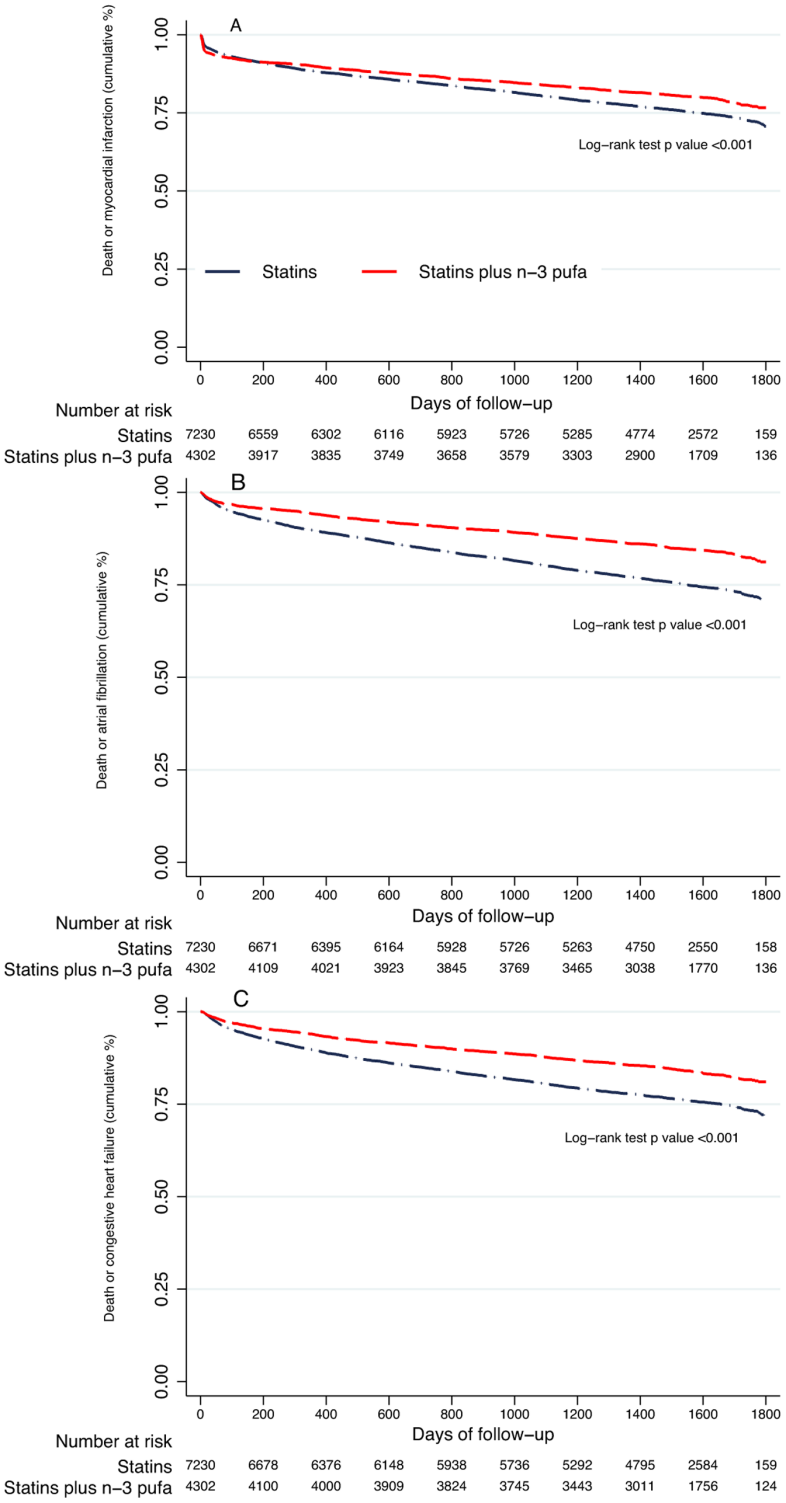
Kaplan-Meier survival curves of the effects of statins plus n-3 PUFA as compared with statins alone on death or myocardial infarction (A), death or atrial fibrillation (B) and death or congestive heart failure (C).

As compared with statins alone, statins plus n-3 PUFA were associated (HR [95% CI], p) with an adjusted higher survival rate (HR = 0.59 [0.52–0.66], p<0.001), survival free of AF (HR = 0.78 [0.71–0.86], p<0.001) and survival free of new heart failure development (HR = 0.81 [0.74–0.88], p<0.001), survival free of stroke (HR = 0.66 [0.59 to 0.74], p<0.001) but not with re-infarction (HR = 0.94 [0.86–1.02], p<0.127) (Table 3, figure 1 and 2).

**Table 3:** Adjusted estimates for major outcomes in patients treated with statins alone or statins plus n-3 PUFA.

Outcomes	Adjusted HR’s (95% CI)	P value
All cause death	0.59 (0.52 to 0.66)	<0.001
Death or myocardial infarction	0.94 (0.86 to 1.02)	0.127
Death or atrial fibrillation	0.78 (0.71 to 0.86)	<0.001
Death or congestive heart failure	0.81 (0.74 to 0.88)	<0.001
Death or stroke	0.66 (0.59 to 0.74)	<0.001

### Sensitivity Analyses

**Figure 3:**
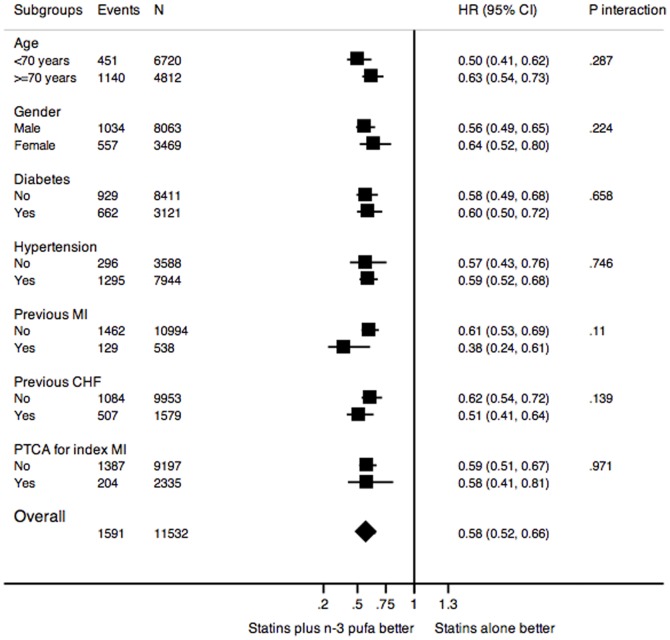
Association between combination therapy and mortality among subsets of patients.

As described for overall study population, combined prescription of statins plus n-3 PUFA was consistently associated with higher survival among various subsets (figure 3).

Particularly, patients who also were prescribed with the recommended secondary prevention treatments (beta blockers, aspirin and ACE inhibitors, n = 7,507 (65.1%) had the same survival advantage with combination therapy as those not exposed to the full preventive regime (HR = 0.60 [0.51–0.70], versus HR = 0.55 [0.45–0.67], p for interaction = 0.283).

### Results of Paired Matched Analysis

Overall, 3,962 patients prescribed with statins plus n-3 PUFA were matched in 1∶1 ratio with the same number of patients prescribed only with statins.

As Table 4 shows baseline characteristics, in-hospital complications and post-discharge prescriptions for the propensity score matched groups were well balanced.

**Table 4:** Baseline characteristics and outcomes in paired-matched population.

Variable		Statins		Statins plus n-3 PUFA		P value*	
N		3,962		3,962		-	
Age, mean (SD)		65.1 (10.2)		65.2 (9.9)		0.29	
Male sex, n (%)		2,909 (73.4)		2,918 (73.6)		0.828	
Diabetes, n (%)		1,094 (27.6)		1,088 (27.5)		0.899	
Hypertension, n (%)		2,643 (66.7)		2,673 (67.5)		0.465	
Previous MI, n (%)		194 (4.9)		184 (4.6)		0.637	
Previous CHF, n (%)		476 (12.0)		476 (12.0)		1.00	
Previous Stroke, n (%)		69 (1.7)		63 (1.6)		0.656	
Previous AF, n (%)		209 (5.3)		219 (5.5)		0.643	
Peripheral vascular disease, n (%)		256 (6.5)		250 (6.3)		0.821	
Previous CABG, n (%)		36 (0.9)		37 (0.9)		1.00	
Previous PTCA, n (%)		174 (4.4)		167 (4.2)		0.742	
COPD, n (%)		402 (10.1)		395 (10.0)		0.815	
Malignancy, n (%)		88 (2.2)		93 (2.3)		0.764	
Depression, n (%)		106 (2.7)		104 (2.6)		0.944	
*In-hospital complications and procedures*							
Heart failure, n (%)		358 (9.0)		354 (8.9)		0.906	
AF, n (%)		164 (4.1)		173 (4.4)		0.650	
Coronary angiography, n (%)		1,852 (46.7)		1,799 (45.4)		0.240	
PTCA, n (%)		834 (21.0)		844 (21.3)		0.805	
CABG, n (%)		67 (1.7)		77 (1.9)		0.447	
*Treatments after discharge*							
Beta-blockers, n (%)		3,305 (83.4)		3,299 (83.3)		0.869	
ACEi/ARB, n (%)		3,539 (89.3)		3,554 (89.7)		0.600	
Clopidogrel, n (%)		1,714 (43.3)		1,667 (42.1)		0.294	
Aspirin, n (%)		3,681 (92.9)		3,666 (92.5)		0.534	
Outcomes							
	Statins		Statins plus n-3 PUFA		Risk ratios (95% CIs)		P value
All cause death	539 (13.6%)		340 (8.6%)		0.63 (0.56 to 0.72)		<0.001
Death or myocardial infarction	848 (21.4)		804 (20.3)		0.95 (0.87 to 1.03)		0.234
Death or atrial fibrillation	805 (20.3)		660 (16.7)		0.82 (0.75 to 0.90)		<0.001
Death or CHF	792 (20.0)		684 (17.3)		0.86 (0.79 to 0.95)		0.002
Death or stroke	662 (16.7)		456 (11.5)		0.65 (0.58 to 73)		<0.001

* All p values are for McNemar’s test.

Results of this analysis confirm main results. Combination therapy was associated with a similar reduction in all tested outcomes, but reinfarction (RR 0.95 [0.87 to 1.03], p = 0.234).

## Discussion

Using a large administrative cohort, this analysis found a reduction in overall mortality with combination therapy and major improvements in most cardiovascular outcomes. In practical terms, the analyses revealed that between 2 to 3 fatal and non-fatal events could be avoided for each hundred patients prescribed annually with the combination therapy as compared with those prescribed with statins, and this advantage appears to be independent of concomitant use of other preventive drugs.

This association was clinically significant for most tested outcomes, including all cause mortality, survival free of heart failure, survival free of atrial fibrillation, survival free of stroke but not re-infarction, suggesting that the addition of n-3 PUFA to the prescription with statins in secondary prevention provided beneficial effects based on others than antiatherosclerotic mechanisms of action.

Although a reduced overall mortality was associated with combination therapy, the mechanism for achieving this effect could not be clearly elucidated from the present analysis. The nature of data arising from administrative datasets made the identification of the cause of death impossible. Yet is tempting to adjudicate the effect on overall mortality as a consequence of a reduction on cardiovascular mortality, this remains speculative. However, a number of prospective studies and systematic reviews [19]–[22] indicate that consumption of fish or fish oil supplements significantly reduces CHD mortality, including fatal myocardial infarction and sudden cardiac death, in populations with and without established CVD. The final common pathway for most cardiac deaths is arrhythmia [2], [23].

However, it should also be noted that recent evidence failed to demonstrate an effect of n-3 PUFA supplementation on several cardiovascular outcomes, including cardiovascular mortality [4], [5], [8]. These trials, however, were each substantially underpowered to detect effects on CHD mortality also, due to lower than expected event rates, the Alpha-Omega trial compared the effects of low-dose EPA+DHA not with placebo, but with a combined control group that received either placebo or active treatment with n-3 ALA [5]. Finally, in both the Alpha-Omega and SU.FOL.OM3 trials were not designed to assess CHD mortality was not the primary outcome and the number of CHD deaths (57 and 40, respectively) were too low to provide a definitive conclusion [4], [8].

The present analysis also found an association between CT and reduced stroke incidence during follow up. Despite a meta-analysis of observational studies suggests that fish consumption reduces risk of ischemic stroke [24], stroke incidence has not been significantly affected in fish oil trials. Reasons for these differing findings remain unclear, with possibilities including residual confounding bias in our analysis but also inadequate statistical power in the trials or insufficient duration of treatment in the RCTs. In any case, our findings were robust, suggestive and supportive of some [24] but not all evidence.

Our findings regarding CT and CHF during follow up are in line with previous animal studies [25], [26] and clinical trials data [11].

Overall results differ in magnitude from those of GISSI-Prevenzione trial, this apparent discrepancies should be cautiously interpreted since there are major differences between baseline characteristics of populations, the studied exposures (i.e. statins versus statins plus n-3 PUFA) and designs [1]. Our findings, that suggests a more pronounced benefit from n-3 PUFA could be a consequence of such differences, but also could indicate residual confounders (see below).

### Study Limitations

The limitations of our study could be considered typical of all observational studies [27]. Administrative databases did not contain detailed clinical information as left ventricular ejection fraction, ventricular volumes and renal function. Hidden biases or inability to account for all factors related to both physicians’ prescription choices and patients’ risk of death, as LDL cholesterol levels at baseline and during the follow-up, might be responsible for the observed differences across groups. However, all analyses were adjusted for multiple potentially confounding variables, including the presence of previous cardiovascular and non-cardiovascular conditions, in-hospital procedures and out-of-hospital treatments. Additionally sensibility analysis including the results obtained in major subgroups and paired-matched analysis were consistent with those obtained in the whole cohort. Propensity score analyses should be interpreted having in mind that these methods only are able to balance for potential measured confounders and administrative data with paucity of covariate information could lead to residual confusion not completely balanced. This limitation could lead to overestimation of the treatment effect magnitude [28]. The consistence between Cox’s proportional hazard models and propensity score matched analyses could indicate the lack of additional benefit from propensity score adjustment in large datasets [29].

Further, it should be acknowledged that, as in most pharmacoepidemiologic studies, exposure time to treatments was measured by filled prescriptions, which may not faithfully measure drug intake. However this bias was minimized using a time-dependent analysis accounting for potential interruptions in pills intake.

Finally, other bias occurring in observational studies like bias from immortal time (that is the time after cohort entry but prior to initiation of treatment) and bias by indication. The first was accounted for using Cox’s proportional models with time-dependent covariates and the second using multivariate analyses and its consistency checked with additional sensitivity analyses.

### Conclusions

Our results suggest that a combination therapy with statins and n-3 PUFA is associated with a relevant benefit in terms of clinical outcomes in patients discharged after MI. Large randomized clinical trials should confirm these results.

## References

[1] GISSI-Prevenzione Investigators (Gruppo Italiano per lo Studio della Sopravvivenza nell'Infarto miocardico) (1999) Dietary supplementation with n-3 polyunsaturated fatty acids and vitamin E after myocardial infarction: results of the GISSI-Prevenzione trial. Lancet 354: 447–455. 10465168

[2] Marchioli R, Barzi F, Bomba E (2002) Chieffo C, Di Gregorio D, et al (2002) Early protection against sudden death by n-3 polyunsaturated fatty acids after myocardial infarction: time-course analysis of the results of the Gruppo Italiano per lo Studio della Sopravvivenza nell'Infarto Miocardico (GISSI)-Prevenzione. Circulation 105: 1897–1903. 11997274 10.1161/01.cir.0000014682.14181.f2

[3] Yokoyama M, Origasa H, Matsuzaki M, Matsuzawa Y, Saito Y, et al (2007) Effects of eicosapentaenoic acid on major coronary events in hypercholesterolaemic patients (JELIS): a randomised open-label, blinded endpoint analysis. Lancet 369: 1090–1098. 17398308 10.1016/S0140-6736(07)60527-3

[4] Galan P, Kesse-Guyot E, Czernichow S, Briancon S, Blacher J, et al.; SU.FOL.OM3 Collaborative Group (2010) Effects of B vitamins and omega 3 fatty acids on cardiovascular diseases: a randomised placebo controlled trial. BMJ 341: c6273 doi: 10.1136/bmj.c6273. 21115589 10.1136/bmj.c6273 PMC2993045

[5] Kromhout D, Giltay EJ (2010) Geleijnse JM; Alpha Omega Trial Group (2010) n-3 fatty acids and cardiovascular events after myocardial infarction. N Engl J Med 363: 2015–2026. 20929341 10.1056/NEJMoa1003603

[6] ORIGIN Trial Investigators, Bosch J, Gerstein HC, Dagenais GR, Díaz R, Dyal L, et al (2012) n-3 fatty acids and cardiovascular outcomes in patients with dysglycemia. N Engl J Med 367: 309–318. 22686415 10.1056/NEJMoa1203859

[7] Kwak SM, Myung SK, Lee YJ (2012) Seo HG; Korean Meta-analysis Study Group (2012) Efficacy of omega-3 fatty acid supplements (eicosapentaenoic acid and docosahexaenoic acid) in the secondary prevention of cardiovascular disease: a meta-analysis of randomized, double-blind, placebo-controlled trials. Arch Intern Med 172: 686–694. 22493407 10.1001/archinternmed.2012.262

[8] Rauch B, Schiele R, Schneider S, Diller F, Victor N, et al (2010) OMEGA, a randomized, placebo-controlled trial to test the effect of highly purified omega-3 fatty acids on top of modern guideline-adjusted therapy after myocardial infarction. Circulation 122: 2152–2159. 21060071 10.1161/CIRCULATIONAHA.110.948562

[9] Davidson MH, Stein EA, Bays HE, Maki KC, Doyle RT, et al (2007) Efficacy and tolerability of adding prescription omega-3 fatty acids 4 g/d to simvastatin 40 mg/d in hypertriglyceridemic patients: an 8-week, randomized, double-blind, placebo-controlled study. Clin Ther 29: 1354–1367. 17825687 10.1016/j.clinthera.2007.07.018

[10] Maki KC, McKenney JM, Reeves MS, Lubin BC, Dicklin MR (2008) Effects of adding prescription omega-3 acid ethyl esters to simvastatin (20 mg/day) on lipids and lipoprotein particles in men and women with mixed dyslipidemia. Am J Cardiol 102: 429–433. 18678300 10.1016/j.amjcard.2008.03.078

[11] Gissi-HF Investigators, Tavazzi L, Maggioni AP, Marchioli R, Barlera S, et al (2008) Effect of n-3 polyunsaturated fatty acids in patients with chronic heart failure (the GISSI-HF trial): a randomised, double-blind, placebo-controlled trial. Lancet 372: 1223–1230. 18757090 10.1016/S0140-6736(08)61239-8

[12] Ayanian JZ (1999) Using administrative data to assess health care outcomes. Eur Heart J 20: 1689–1691. 10562474 10.1053/euhj.1999.1823

[13] Monte S, Macchia A, Pellegrini F, Romero M, Lepore V, et al (2006) Antithrombotic treatment is strongly underused despite reducing overall mortality among high-risk elderly patients hospitalized with atrial fibrillation. Eur Heart J 27: 2217–2223. 16935869 10.1093/eurheartj/ehl208

[14] WHO Collaborating Centre for Drug Statistics Methodology (2003) ATC Index with DDDs. Oslo, Norway: WHO.

[15] Kalbfleisch JD, Prentice RL (1980) The Statistical Analysis of Failure Time Data. New York: John Wiley.

[16] Therneau TM, Grambsch PM (2000) Modeling Survival Data: Extending the Cox Model. New York: Springer.

[17] Collett D (1994) Modelling Survival Data in Medical Research. London: Chapman and Hall.

[18] Cox DR (1972) Regression models and life-tables. Journal of the Royal Statistical Society B 34: 187–220.

[19] Mozaffarian D, Rimm EB (2006) Fish intake, contaminants, and human health: evaluating the risks and the benefits. JAMA 296: 1885–1899. 17047219 10.1001/jama.296.15.1885

[20] León H, Shibata MC, Sivakumaran S, Dorgan M, Chatterley T, et al (2008) Effect of fish oil on arrhythmias and mortality: systematic review. BMJ 337: a2931 doi: 10.1136/bmj.a2931. 19106137 10.1136/bmj.a2931 PMC2612582

[21] Yamagishi K, Iso H, Date C, Fukui M, Wakai K, et al (2008) Fish, omega-3 polyunsaturated fatty acids, and mortality from cardiovascular diseases in a nationwide community-based cohort of Japanese men and women the JACC (Japan Collaborative Cohort Study for Evaluation of Cancer Risk) Study. J Am Coll Cardiol 52: 988–996. 18786479 10.1016/j.jacc.2008.06.018

[22] Kromhout D, Geleijnse JM, de Goede J, Oude Griep LM, Mulder BJ, et al (2011) n-3 fatty acids, ventricular arrhythmia-related events, and fatal myocardial infarction in postmyocardial infarction patients with diabetes. Diabetes Care 34: 2515–2520. 22110169 10.2337/dc11-0896 PMC3220851

[23] Leaf A, Albert CM, Josephson M, Steinhaus D, Kluger J, et al (2005) Prevention of fatal arrhythmias in high-risk subjects by fish oil n-3 fatty acid intake. Circulation 112: 2762–2768. 16267249 10.1161/CIRCULATIONAHA.105.549527

[24] Harris WS, Mozaffarian D, Lefevre M, Toner CD, Colombo J, et al (2009) Towards establishing dietary reference intakes for eicosapentaenoic and docosahexaenoic acids. J Nutr 139: 804S–819S. 19244379 10.3945/jn.108.101329 PMC6459058

[25] Duda MK, O'Shea KM, Tintinu A, Xu W, Khairallah RJ, et al (2009) Fish oil, but not flaxseed oil, decreases inflammation and prevents pressure overload-induced cardiac dysfunction. Cardiovasc Res 81: 319–327. 19015135 10.1093/cvr/cvn310 PMC2721645

[26] Teng LL, Shao L, Zhao YT, Yu X, Zhang DF, et al (2010) The beneficial effect of n-3 polyunsaturated fatty acids on doxorubicin-induced chronic heart failure in rats. J Int Med Res 38: 940–948. 20819430 10.1177/147323001003800320

[27] Byar DP (1991) Problems with using observational databases to compare treatments. Stat Med 10: 663–666. 2057663 10.1002/sim.4780100417

[28] Austin PC, Mamdani MM, Stukel TA, Anderson GM, Tu JV (2005) The use of the propensity score for estimating treatment effects: administrative versus clinical data. Stat Med 24: 1563–1578. 15706581 10.1002/sim.2053

[29] Biondi-Zoccai G, Romagnoli E, Agostoni P, Capodanno D, Castagno D, et al (2011) Are propensity scores really superior to standard multivariable analysis? Contemp Clin Trials 32: 731–740 doi: 10.1016/j.cct.2011.05.006. 21616172 10.1016/j.cct.2011.05.006

