# Correction: Exploratory Analysis on the Use of Statins with or without n-3 PUFA and Major Events in Patients Discharged for Acute Myocardial Infarction: An Observational Retrospective Study

**DOI:** 10.1371/journal.pone.0126303

**Published:** 2015-04-17

**Authors:** The PLOS ONE Staff

There is an error in Table 4. The Risk ratios for the last row, “Death or stroke” should read as follows: 0.65 (0.58 to 0.73).

Please find the complete, correct Table 4 here:

**Table 4:** Baseline characteristics and outcomes in paired-matched population.

Variable	Statins		Statins plus n-3 PUFA		P value*	
N	3,962		3,962		-	
Age, mean (SD)	65.1 (10.2)		65.2 (9.9)		0.29	
Male sex, n (%)	2,909 (73.4)		2,918 (73.6)		0.828	
Diabetes, n (%)	1,094 (27.6)		1,088 (27.5)		0.899	
Hypertension, n (%)	2,643 (66.7)		2,673 (67.5)		0.465	
Previous MI, n (%)	194 (4.9)		184 (4.6)		0.637	
Previous CHF, n (%)	476 (12.0)		476 (12.0)		1.00	
Previous Stroke, n (%)	69 (1.7)		63 (1.6)		0.656	
Previous AF, n (%)	209 (5.3)		219 (5.5)		0.643	
Peripheral vascular disease, n (%)	256 (6.5)		250 (6.3)		0.821	
Previous CABG, n (%)	36 (0.9)		37 (0.9)		1.00	
Previous PTCA, n (%)	174 (4.4)		167 (4.2)		0.742	
COPD, n (%)	402 (10.1)		395 (10.0)		0.815	
Malignancy, n (%)	88 (2.2)		93 (2.3)		0.764	
Depression, n (%)	106 (2.7)		104 (2.6)		0.944	
*In-hospital complications and procedures*						
Heart failure, n (%)	358 (9.0)		354 (8.9)		0.906	
AF, n (%)	164 (4.1)		173 (4.4)		0.650	
Coronary angiography, n (%)	1,852 (46.7)		1,799 (45.4)		0.240	
PTCA, n (%)	834 (21.0)		844 (21.3)		0.805	
CABG, n (%)	67 (1.7)		77 (1.9)		0.447	
*Treatments after discharge*						
Beta-blockers, n (%)	3,305 (83.4)		3,299 (83.3)		0.869	
ACEi / ARB, n (%)	3,539 (89.3)		3,554 (89.7)		0.600	
Clopidogrel, n (%)	1,714 (43.3)		1,667 (42.1)		0.294	
Aspirin, n (%)	3,681 (92.9)		3,666 (92.5)		0.534	
Outcomes						
	Statins	Statins plus n-3 PUFA		Risk ratios (95% CIs)		P value
All cause death	539 (13.6%)	340 (8.6%)		0.63 (0.56 to 0.72)		<0.001
Death or myocardial infarction	848 (21.4)	804 (20.3)		0.95 (0.87 to 1.03)		0.234
Death or atrial fibrillation	805 (20.3)	660 (16.7)		0.82 (0.75 to 0.90)		<0.001
Death or CHF	792 (20.0)	684 (17.3)		0.86 (0.79 to 0.95)		0.002
Death or stroke	662 (16.7)	456 (11.5)		0.65 (0.58 to 0.73)		<0.001

*All p values are for McNemar’s test
